# AN UPDATE ON THE RETURN OF THE GERIATRICS ACADEMIC CAREER AWARDS PROGRAM

**DOI:** 10.1093/geroni/igz038.1360

**Published:** 2019-11-08

**Authors:** Leland Waters, Maura Brennan

**Affiliations:** 1 Virginia Commonwealth University, Richmond, Virginia, United States; 2 University of Massachusetts Medical School-Baystate, Springfield, Massachusetts, United States

## Abstract

The Health Resources and Services Administration recently announced a notice of funding opportunity (NOFO) for the Geriatrics Academic Career Award Program (GACA). The purpose of the GACA program is to support the career development of individual junior faculty in geriatrics as academic geriatrics specialists and to provide clinical training in geriatrics, including the training of interprofessional teams of health care professionals. The GACA program is a mentorship model where the awardee is guided through a faculty career development plan, with required project deliverables, over the course of four years. The GACA supported 222 junior faculty members between 1998 and 2006, when it was discontinued. This initial NOFO provides funding for up to 26 awardees in the schools of allopathic medicine, osteopathic medicine, nursing, social work, psychology, dentistry, pharmacy, and allied health. This presentation will provide an up-to-date overview of the program with awardee demographics and project deliverables.

